# Prevalence of and factors associated with dental service utilization among early elderly in Lithuania

**DOI:** 10.1186/s12913-021-07388-y

**Published:** 2022-01-02

**Authors:** Sergei N. Drachev, Alina Puriene, Jolanta Aleksejuniene, Indre Stankeviciene, Lina Stangvaltaite-Mouhat

**Affiliations:** 1 Department of Clinical Dentistry, Faculty of Health Sciences, UiT The Arctic University of Norway, N-9037 Tromsø, Norway; 2 Department of Prosthodontics, Northern State Medical University, Arkhangelsk, Russia; 3 Institute of Dentistry, Faculty of Medicine, Vilnius University, Vilnius, Lithuania; 4 Department of Preventive and Community Dentistry, Faculty of Dentistry, The University of British Columbia, Vancouver, Canada; 5 Oral Health Centre of Expertise in Eastern Norway, Oslo, Norway

**Keywords:** Dental service utilization, Early elderly, Andersen’s behavioural model, Lithuania

## Abstract

**Background:**

There is no recent information about dental service utilization (DSU) among elderly in Lithuania. We examined DSU and its associated factors in Lithuanian early elderly based on the Andersen’s behavioural model.

**Methods:**

The cross-sectional study conducted in 2017–2019 included a nationally representative stratified sample of 370 Lithuanian early elderly aged 65–74 years (response rate of 54.5%). Information on predisposing factors (age, sex, nationality and education), enabling factor (residence), need-based factors (status of teeth, oral pain or discomfort, and dry mouth), general health, personal health practices and perceived stress was obtained from a structured, self-administered questionnaire. Clinically-assessed need-based factors included number of missing teeth and dental treatment need. Multivariable Poisson regression with robust variance estimates was used.

**Results:**

A total of 239 study participants (64.6%) reported a dental visit during the last year and 338 (91.4%) needed dental treatments. A higher level of education (adjusted prevalence ratio [aPR] = 1.21, 95% confidence interval [CI]:1.04–1.40), pain or discomfort in teeth/mouth (aPR = 1.35, 95%CI: 1.13–1.62) and lower number of missing teeth (aPR = 0.99, 95%CI: 0.98–1.00) were associated with DSU.

**Conclusions:**

Even though majority of early elderly needed dental treatments, only two-thirds visited a dentist during the last year. Predisposing and need-based factors were significant predictors of having a dental visit in the last year. A national oral health program for Lithuanian elderly with the focus on regular preventive dental check-ups is needed. More studies, both quantitative and qualitative, are warranted to investigate in depth the barriers for DSU among elderly in Lithuania.

## Background

Oral health in elderly is an important public health issue. Despite significant progress in preventive measures that promote oral health in children, oral diseases and conditions such as dental caries, periodontal diseases, hyposalivation, xerostomia, and oral cancer remain prevalent and often underdiagnosed and untreated in elderly. These oral problems may lead to pain and discomfort, tooth loss, local and systemic inflammation, impairment of a person’s functional, social and psychological well-being. Availability and accessibility of dental care to elderly, their willingness to seek it are important to identify oral health problems at early stages and provide necessary preventive and curative measures [1].

Many studies examining dental service utilization (DSU) and its determinants applied the Andersen’s behavioural model of healthcare use [2–6]. This model originally developed in 1960s evolved over time although the fundamental components of the model have not substantially changed [7]. The model includes individual and contextual-level components such as *predisposing factors* (for ex., age, sex, education, cultural norms), *enabling factors* (for ex., income and wealth, insurance, transportation, hospital and dentist density), and *need-based factors* (for ex., level of injury or disease assessed by individual or physician/dentist). Importantly, systemic diseases and medications as sources of hyposalivation and xerostomia in elderly may also cause deterioration in oral health and quality of life and lead to seek dental care [1, 8]. In a revised Andersen’s model, *personal health practices* (for ex., diet, exercise, alcohol, tobacco, frequency of tooth-brushing) were added [5–7, 9]. Moreover, *psychosocial factors* may determine health and dental service use [10]. Indeed, in Germany frequent attendance in primary care was associated with higher perceived stress and less self-efficacy, self-esteem, life satisfaction, and self-regulation in people aged 40+ years [11]. Despite that psychosocial factors may play an important role in DSU, studies examining such relationships in elderly are scarce. A Brazilian study did not find a significant association between use of dental service in previous 3 years and depressive symptoms [6]. Interestingly, a systematic 2021 review reported that the components of Andersen’s behavioural model are more likely to be associated with DSU in children, whereas predisposing, enabling, and need-based factors have not been consistently associated with DSU in adults [12]. In adults, a link between increased dental service use and higher education (predisposing factor) was found [12].

Lithuania is one of the Baltic States in Northern Europe with a total population of 2.8 million in 2020, of whom 71% are living in urban areas [13]. An oral health survey conducted in 1997/1998, more than 20 years ago, showed that only 42% of Lithuanian elderly aged 65–74 years visited a dentist within the past year [14]. Women, persons living in urban areas, and persons who had more natural teeth and positive attitudes to personal dental care used dental service more frequently. A higher level of education and experiencing oral problems were significant determinants of DSU. In addition, the researchers reported that accessibility to dental care was limited in rural areas due to a lower number of practicing dentists [14]. Importantly, the study was performed when the Lithuanian dental health care system was transitioning from centralized Soviet Union model to a two-tier delivery model including both public and private dentistry [14, 15]. Moreover, profound macroeconomic changes that occurred in Lithuania in 2004–2012 had a large impact on DSU [16]. Indeed, a significant increase in dental visits among Lithuanian adults aged 20–64 years in 2004–2008 was followed by a significant decline in 2010. A strong positive socioeconomic gradient in DSU was also observed [16]. However, there is no recent information available on DSU and its related factors in Lithuanian elderly.

**Fig. 1:**
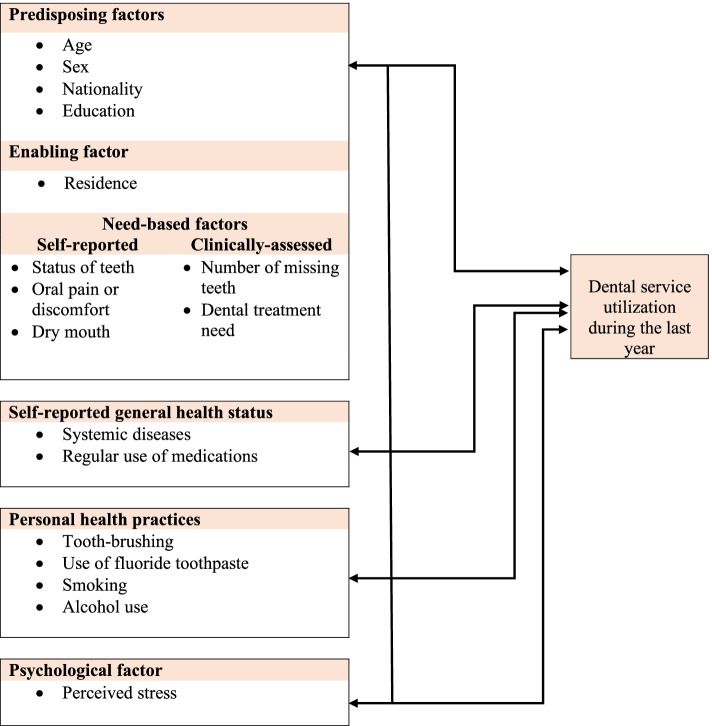
Dental service utilization model of the present study adapted from Andersen, 2008 [7]

The aim of the study was to examine DSU and its associated factors in a nationally representative sample of Lithuanian early elderly aged 65–74 years using the Andersen’s behavioural model as the theoretical framework. The DSU model of the present study is presented in Fig. 1.

## Methods

### Study design, population, and sampling

The current cross-sectional study was carried out as a part of the Lithuanian National Oral Health Survey conducted in 2017–2019. The study was approved by the Lithuanian Bioethical Committee (No. 158200–17–920-426) and the Personal Data Protection Authority (No. 2R-4077). Altogether, 679 early elderly people aged 65–74 years from the five largest Lithuanian cities and 10 randomly selected peri-urban/rural areas, one in each of 10 Lithuanian counties, were extracted from the patient lists at primary health care institutions and invited to participate in the study. A total of 370 participants (response rate of 54.5%) completed a structured self-administered questionnaire and underwent a clinical dental examination. More details on sampling have been published elsewhere [17, 18].

### Data collection

The World Health Organization (WHO) Oral Health Questionnaire for Adults [19] was used. The English version of the questionnaire was translated into an official Lithuanian language and two minority languages (Russian and Polish) using the forward-backward translation technique [17, 18]. Before the study began, the questionnaire was pretested in 10 adults who did not participate in the main study. In addition, self-reported information about dry mouth, systemic diseases, regular use of medications, and perceived stress was gathered. All study participants underwent a clinical dental examination performed in accordance with the WHO recommendations [19]. All permanent teeth including third molars were taken into consideration. One calibrated dentist (I.S.) performed clinical examinations, and the level of intra-examiner agreement was satisfactory [17].

### Dependent variable

The variable “DSU during the last year” was the study’s outcome. Study participants reported when they visited a dentist last time. Responses included (1) less than 6 months ago, (2) 6–12 months ago, (3) more than 1 year ago, but less than 2 years ago, (4) 2 years ago or more, but less than 5 years ago, (5) 5 years ago or more, (6) never received dental care. For analysis, original responses of the “DSU during the last year” were dichotomized into yes (1,2) or no (3–6).

### Independent variables

This set of variables consisted of predisposing, enabling, and need-based factors, self-reported general health status, personal health practices, and psychological factors. Predisposing factors included two age groups (65–69/70–74 years), sex, nationality (Lithuanian /Russian /Belorussian/ Ukrainian/Polish/other), and education (no education /less than primary/primary/secondary/higher non-university/university: Bachelor degree or higher). For analysis, the variable “education” was categorized into: less than university and university. Residence (urban/peri-urban/rural) was considered as an enabling factor. Information on need-based factors was gathered from both the questionnaire and clinical dental examination. Study participants were asked to report about their teeth status on a 6-point scale: (1) excellent, (2) very good, (3) good, (4) average, (5) poor, (6) very poor. For analysis, three categories were used: good (1–3), average (4), and poor (5,6). The question also included the response option “do not know”. If that response was chosen (*n* = 27), this data was considered missing. The variable “experienced pain or discomfort in teeth or mouth in the last year” was dichotomized into yes and no/do not know. Information on dry mouth (never/sometimes/often/always) was also collected. Study participants reporting dry mouth sometimes, often or always were categorized as having, others as not having dry mouth. Information on number of missing teeth due to caries or for any other reason and dental treatment need was obtained from the clinical dental examination. In accordance with the WHO recommendations for oral health surveys [19], the variable “dental treatment need” had the following response options: (0) no treatment, (1) preventive or routine treatment, (2) prompt treatment including scaling, (3) urgent treatment due to local oral pain or infection, (4) need to be referred for a comprehensive evaluation or medical/dental treatment (systemic condition). For analysis, this variable was categorized into no (0) and yes (1–4).

Questions on self-reported general health status included presence of systemic diseases (yes/no) and taking medications on regular basis (yes/no). Tooth-brushing frequency, using fluoride toothpaste, cigarette smoking, and alcohol use were considered as personal health practices. For analysis, tooth-brushing frequency was dichotomized into less than twice a day (never/once a month/2–3 times a month/once a week/2–6 times a week/once a day) and twice a day or more frequently. The variable “using fluoride toothpaste” was categorized as yes and no/do not know. Cigarette smoking was assessed as no (never) and yes (seldom, several times a month, once a week, several times a week, every day). Moreover, study participants were asked how much alcohol they usually drank per day in the last month. Responses were: (0) less than 1 unit, (1) 1 unit, (2) 2 units, (3) 3 units, (4) 4 units, (5) 5 units and more, (6) did not drink alcohol in the last month. For analysis, this variable included two categories: yes (0–5) and no (6).

Perceived stress was considered as a psychological factor and measured by the PSS-10 (Perceived Stress Scale 10). The translation of the PSS-14, which contains the same 10 items as PSS-10 and four additional items, into Lithuanian was previously done [20]. Given that the PSS-10 was found to have superior psychometric properties than the PSS-14 [21], we selected the PSS-10 items from the translated PSS-14 version. Study participants were asked to report how often they found 10 different situations in their life as stressful during the last month. This scale included 5-point Likert scale responses: 0 = never, 1 = almost never, 2 = sometimes, 3 = fairly often, 4 = very often. Four items (4,5,7,8) were recoded because of the reverse scoring, and a total PSS-10 score was calculated as the sum of all PSS-10 items. The total PSS-10 scores were grouped into three perceived stress groups as follows: low (scores 0–13), moderate (scores 14–26), and high (scores 27–40) [22, 23]. The internal consistency of the PSS-10 scale was acceptable as indicated by the Cronbach’s alpha of 0.75.

### Statistical analyses

The Chi-square test was used to compare proportions of elderly people who visited a dentist during the last year and who did not report such visits among categories of independent variables. The number of missing teeth between the elderly who had and the ones who did not have a dental visit during the last year was compared employing the Mann-Whitney U test.

Multivariable Poisson regression with robust variance estimates was used [24], with the binary dependent variable coded as 0 = did not visit a dentist during the last year, 1 = visited a dentist during the last year, and choosing the independent variables with *p*-values < 0.15 from the bivariable analyses. The results are presented as adjusted prevalence ratios (aPRs) with 95% confidence intervals (CIs). Significance level for all tests was set up at α = 5%.

The most of variables used in analyses had missing values that varied from 0.3 to 7.8%. Of all, 23.8% of the respondents had missing values in PSS-10 scale. To mitigate a potential bias, the missing values in the items of the PSS-10 scale were substituted by the median for any missing item. The analysis was repeated with the substituted data, and no significant differences were found between the two analyses. Final multivariable analysis was based on the complete data i.e. with no missing values.

All analyses were performed using IBM SPSS Statistics version 26.0 (IBM Corp., Armonk, NY, USA) and STATA version 15.0 (StataCorp, College Station, Texas, USA).

## Results

A total of 370 persons aged 64–75 years were included in the statistical analyses, of which 239 (64.6%) reported a dental visit during the last year, 122 (33.0%) did not report such visit, and 9 (2.4%) did not answer. The prevalence of DSU was 68.6 and 65.5% in early elderly aged 65–69 and 70–74 years, respectively.

Sample characteristics as well as findings from the bivariable analyses for the DSU outcome are presented in Tables 1, 2 and 3. The majority of study participants were Lithuanian women, had an education that was lower than university and resided in urban areas. From all predisposing and enabling factors, a university education was found to be the only significant factor associated with DSU, *p* = 0.018 (Table 1).

**Table 1:** Predisposing and enabling factors in association with dental service utilization

		Dental visit in the last year		***p*** values*
Independent variables	n (%)	Yes, n (%)	No, n (%)	
**Age groups**				
65–69 years	193 (52.2)	127 (53.1)	63 (51.6)	0.824
70–74 years	177 (47.8)	112 (46.9)	59 (48.4)	
**Sex**				
Males	116 (31.4)	69 (28.9)	42 (34.4)	0.335
Females	254 (68.6)	170 (71.1)	80 (65.6)	
**Nationality**				
Lithuanian	307 (83.0)	202 (85.2)	98 (81.0)	0.363
Non-Lithuanian**	60 (16.2)	35 (14.8)	23 (19.0)	
Missing	3 (0.8)			
**Education**				
Less than university	263 (71.1)	163 (71.2)	97 (82.9)	0.018
University	87 (23.5)	66 (28.8)	20 (17.1)	
Missing	20 (5.4)			
**Residence**				
Urban	269 (72.7)	172 (72.3)	91 (74.6)	
Peri-urban	55 (14.9)	37 (15.5)	17 (13.9)	0.918
Rural	45 (12.2)	29 (12.2)	14 (11.5)	
Missing	1 (0.3)			

Total numbers across categories of the variable “Dental visit” differ due to missing data

* Chi-square test

** Non-Lithuanian included Russian/Belorussian/Ukrainian/Polish/other

**Table 2:** Need-based factors and self-reported general health status in association with dental service utilization

Independent variables	n (%)	Dental visit in the last year		***p*** values*
		Yes, n (%)	No, n (%)	
**Self-reported status of teeth**				0.077
Good	45 (12.2)	27 (11.7)	17 (15.6)	
Average	153 (41.4)	95 (41.3)	55 (50.5)	
Poor	145 (39.2)	108 (47.0)	37 (33.9)	
Missing	27 (7.3)			
**Experienced pain/discomfort in teeth or mouth in the last year**				< 0.001
Yes	214 (57.8)	159 (68.5)	54 (45.8)	
No/do not know	144 (38.9)	73 (31.5)	64 (54.2)	
Missing	12 (3.2)			
**Self-reported dry mouth**				0.332
No	261 (70.5)	164 (68.6)	90 (73.8)	
Yes	109 (29.5)	75 (31.4)	32 (26.2)	
**Number of missing teeth**	**n (%)**	**mean (sd)**	**mean (sd)**	
	15.1 (8.6)	14.2 (7.7)	16.6 (9.8)	0.051
**Dental treatment need**	**n (%)**	**n (%)**	**n (%)**	0.008
No	32 (8.6)	13 (5.4)	17 (13.9)	
Yes	338 (91.4)	226 (94.6)	105 (86.1)	
**Self-reported systemic diseases**				0.889
No	72 (19.5)	46 (21.0)	26 (22.0)	
Yes	269 (72.7)	173 (79.0)	92 (78.0)	
Missing	29 (7.8)			
**Self-reported regular medications**				1.000
No	76 (20.5)	50 (22.0)	26 (22.2)	
Yes	271 (73.2)	177 (78.0)	91 (77.8)	
Missing	23 (6.2)			

Total numbers across categories of the variable “Dental visit” differ due to missing data

sd standard deviation

^*^p-values were calculated using Chi-square test for categorical variables and Mann-Whitney U for the variable “number of missing teeth”

**Table 3:** Personal health practices and perceived stress in association with dental service utilization

Independent variables	n (%)	Dental visit in the last year		*p*-values*
		Yes, n (%)	No, n (%)	
Frequency of tooth-brushing**				0.411
Less than twice a day	178 (51.7)	116 (50.2)	59 (55.7)	
Twice a day or more	163 (47.4)	115 (49.8)	47 (44.3)	
Missing	3 (0.9)			
Using fluoride toothpaste**				0.158
Yes	152 (44.2)	96 (41.6)	53 (50.0)	
No/do not know	192 (55.8)	135 (58.4)	53 (50.0)	
**Cigarette smoking**				0.220
No	313 (84.6)	208 (92.9)	102 (88.7)	
Yes	29 (7.8)	16 (7.1)	13 (11.3)	
Missing	28 (7.6)			
**Alcohol use**				0.736
No	180 (48.6)	113 (47.9)	59 (50.0)	
Yes	183 (49.5)	123 (52.1)	59 (50.0)	
Missing	7 (1.9)			
**Perceived stress**				0.599
Low	93 (25.1)	65 (35.1)	26 (29.2)	
Moderate	171 (46.2)	109 (58.9)	57 (64.1)	
High	18 (4.9)	11 (6.0)	6 (6.7)	
Missing	88 (23.8)			

Total numbers across categories of the variable “Dental visit” differ due to missing data

* Chi-square test

** Edentulous individuals (*n* = 26) were excluded

**Table 4:** Predisposing and need-based factors associated with dental service utilization (multivariable Poisson regression model)

Predictors	aPR* (95% CI)	*p*-values
**Education**		
Less than university	Reference	
University	1.21 (1.04–1.40)	0.015
**Self-reported state of teeth**		
Good	Reference	
Average	0.97 (0.74–1.28)	0.841
Poor	1.14 (0.87–1.50)	0.332
**Experienced pain or discomfort in teeth or mouth in the last year**		
No/do not know	Reference	
Yes	1.35 (1.13–1.62)	0.001
**Number of missing teeth**	0.99 (0.98–1.00)	0.046
**Dental treatment need**		
No	Reference	
Yes	1.20 (0.74–1.92)	0.463

^*^aPR adjusted prevalence ratio, *CI* confidence interval; *aPRs* are presented from multivariable Poisson regression model with robust variance estimates (*n* = 319); dependent variable: Dental visit in the last year (0 = No, 1 = Yes)

Overall, self-reported status of teeth in the early elderly was average or poor (Table 2). More than half of the study participants experienced pain or discomfort in their teeth or mouth in the last year, and 30% reported dry mouth. The mean number of missing teeth was 15.1, and 7.0% of early elderly were edentulous. The majority needed dental treatment, reported systemic diseases and took medications regularly. Respondents who experienced pain or discomfort in teeth or mouth in the last year utilized dental service more frequently than those who did not experience pain or discomfort or did not know about it. Study participants who visited a dentist during the last year had fewer missing teeth than those who did not report such visits, although this difference was marginally significant. There was a significant association between dental treatment need and having a dental visit in the last year.

In the study sample, 47.4% of early elderly brushed their teeth twice a day or more frequently, and 44.2% used a toothpaste with fluoride. Almost 85% of respondents did not smoke cigarettes, while there was a similar number of respondents who consumed (49.5%) or did not consume (48.6%) alcohol during the last month. The mean PSS-10 score was 16.4, and almost half of the study participants reported moderate level of perceived stress. No statistically significant differences were observed in DSU during the last year between different categories of personal health practices and perceived stress (Table 3).

Multivariable analysis showed that experiencing pain or discomfort in teeth or mouth in the last year was the strongest predictor of having a dental visit. A higher level of education and a lower number of missing teeth were also significant predictors of DSU (Table 4).

## Discussion

This is the first study in Lithuania in last 20 years to assess dental service utilization among early elderly. The response rate of 54.5% was comparable with the rate observed in a previous Lithuanian study conducted in 1997/1998 (55.0%) [14]. A sample covering country-wide geographical locations was selected. Moreover, 68.6% of our participants were females, which quite accurately represented the sex distribution of all permanent inhabitants aged 65–74 years in Lithuania, where women constituted 61.9% of all inhabitants in 2018 [25]. The study showed that two-thirds of Lithuanian early elderly visited a dentist during the last year. One predisposing factor (a higher level of education) and two need-based factors (experiencing pain or discomfort in teeth or mouth and lower number of missing teeth) were significant predictors of having a dental visit in the last year.

Some limitations should be taken into consideration when interpreting the study results. A cross-sectional study design does not allow any causal inferences. We considered only one enabling factor (residence), as information about other potential enabling factors such as perceived access to dental care, dental anxiety, and transportation or travel time was not available. Moreover, no economic determinants of DSU [26] such as income, wealth, family affluence, and poverty have been examined. In addition, under-representation of rural population aged 65–74 years (12.2% in the current study vs. 32.6% in the 2018 Lithuanian report [25]) may limit the generalizability of our findings at the national level. Although we calculated Cronbach’s alpha for PSS-10, other approaches to validate PSS-10 in the study sample were not implemented. Information about dental visits, general health status, personal health practices, and psychological factors was self-reported, therefore the potential recall bias cannot be excluded. Moreover, social desirability bias in answering some questions (tooth-brushing frequency, alcohol use, cigarette smoking) cannot be ruled out.

In our study, the prevalence of dental visit in the last year among Lithuanian early elderly (64.9%) was higher than the one (42.0%) reported in the previous 1997/1998 Lithuanian study [14]. This time-related change may be partly explained by the substantial changes in Lithuanian dental health care system after the country regained its independence in 1990. Since that time, public free-of-charge dental care delivery system gradually transformed into a two-tier dental care delivery model including both public and private dentistry [14, 15]. Adults receiving dental treatments in public and private clinics funded by the National Health Insurance Fund (NHIF) have to cover only the cost of the dental materials, while dental patients in private clinics not funded by NHIF need to cover the full cost of dental treatments [27]. One may assume that patients have not been used to pay for their dental treatment, which might result in a lower DSU in 1997/1998. Twenty years later, more elderly might have accepted the current dental health care system and better understand their responsibility for their own dental health, which, to some extent, could lead to increased use of professional dental services. Nevertheless, despite increasing pensions and that dental care for Lithuanian elderly is partially subsidized by NHIF, this compensation is still insufficient, consequently many Lithuanian elderly cannot cover the cost of their dental treatments from their retirement money [28, 29]. In 2006–2007, a considerable variation in dental attendance in the past year among older adults was found in other European countries [30]. The prevalence of DSU among elderly aged 65–69 and 70–74 years in the present study (68.6 and 65.5%) was lower than in Denmark (83.9 and 71.4%), Sweden (84.0 and 85.9%), Germany (78.1 and 74.0%), and Switzerland (73.8 and 75.1%), but it was higher than in France (46.0 and 52.3%), Greece (40.0 and 28.7%), Italy (39.5 and 30.5%), and Poland (19.5 and 15.8%) [30]. Direct comparison of our results with data obtained from other countries must be done with caution due to differences in the professional dental care infrastructure, health insurance systems, and subsidy for dental care costs [31]. Moreover, some elderly studies used different outcomes for dental care utilization, for example having or not a dental visit during the past 3 years [6] or 5 years [4]. These researchers assumed 12 months to be a too short period to perform analysis at this age group because elderly do not use dental service so often, also probably due to a relatively high level of edentulism [6]. Indeed, dental caries and periodontal diseases are not relevant for edentulous subjects, but there are other oral diseases and conditions such as oral cancer, hyposalivation and xerostomia, problems with protheses that should be diagnosed at the earliest stage and, therefore, edentulous people are also recommended to visit a dentist regularly [1]. Although there is no a universal cut-off to assess time since the last dental service use among elderly [6], in our study we applied 1 year as the cut-off.

In the present study, experiencing pain and discomfort in teeth or mouth in the last year was the strongest significant factor associated with DSU. The similar results were reported in Korean adults aged 25–79 years [3] and Brazilian elderly aged 65 years or older [32]. One may assume that elderly use dental service to seek dental treatment, but not for regular preventive check-ups. The fact that more than 90% of early elderly Lithuanians needed dental treatments support this explanation. Dental problems were also found to be a dominant reason for DSU in Lithuanian university employees aged 35–44 years [33]. The researchers found a very low rate of dentists’ recalls as the main reason for dental attendance among middle-aged adults. One may assume that the recall practice has not been adopted for elderly Lithuanians as well, and dentists prefer to solve existing oral health problems rather than to focus on preventive strategies. Therefore, a national oral health program focusing on prevention of oral diseases in elderly is needed in Lithuania.

Our study participants who visited a dentist in the last year had fewer missing teeth than those who did not report such visits. This finding is in line with previous reports, which showed that absence of teeth is considered an important factor of dental service non-utilization [6, 31, 34, 35]. More efforts should be taken to change this trend, and regular dental check-ups regardless of the presence of natural teeth should be encouraged in Lithuanian early elderly.

In the present study, more educated elderly had a higher prevalence of DSU, and this is in agreement with other studies [3, 4, 6, 30, 32]. This finding may be explained by that fact that more educated people are more aware on detrimental factors for oral health and have a higher level of motivation to seek dental care. Nevertheless, in our study personal health practices as well as having systemic diseases, taking medications regularly, and perceived stress were not associated with DSU. In contrast, a Brazilian study reported that elderly with poor self-assessed general health and former alcohol consumption had a lower prevalence of DSU [6].

Importantly, the same factors (experiencing problems in mouth, higher level of education, more of natural teeth left) that were associated with DSU in the previous 1997/1998 Lithuanian study were also significant in our study [14]. Nevertheless, although 20 years ago people living in urban areas used dental service more frequently than those living in rural areas [14], residence was a non-significant factor in our study, and this finding was unexpected. On one hand, the number of dentists in Lithuania has increased from 6.9 dentists in 2000 to 10.0 dentists per 10,000 inhabitants in 2017 [36]. On the other hand, the distribution of dentists and especially dental specialists in Lithuania was found to be uneven – specialists mainly practice in cities and suburbs, unsurprisingly inhabitants of rural areas have a limited access due to a shortage of dentists [15]. In addition, decreasing number of outpatient dental clinics in rural areas and poor public transportation to reach dentists in the district clinics limit access to professional dental care for rural Lithuanians [37]. Therefore, greater financial support/subsidizing from the state and better access to dental service are also important to increase the prevalence of DSU among elderly in Lithuania. It cannot be ruled out that under-representation of the rural population in our study did not allow us to find the significant differences in DSU among elderly living in urban and rural areas.

## Conclusions

Even though majority of early elderly needed dental treatments, only two-thirds visited a dentist during the last year. Predisposing and need-based factors were significant predictors of having a dental visit in the last year. A national oral health program for Lithuanian elderly with the focus on regular preventive dental check-ups is needed. More studies, both quantitative and qualitative, are warranted to investigate in depth the barriers for DSU among elderly in Lithuania.

## Data Availability

The dataset used in the current study is available from the corresponding author on a reasonable request.

## Abbreviations

aPR: Adjusted prevalence ratio
CI: Confidence interval
DSU: Dental service utilization
PSS-10: Perceived Stress Scale 10
SD: Standard deviation
WHO: World Health Organization

## References

[1] Kossioni AE, Hajto-Bryk J, Maggi S, McKenna G, Petrovic M, Roller-Wirnsberger RE, Schimmel M, Tamulaitienè M, Vanobbergen J, Müller F (2018). An expert opinion from the European College of Gerodontology and the European geriatric medicine society: European policy recommendations on Oral health in older adults. J Am Geriatr Soc.

[2] Herkrath FJ, Vettore MV, Werneck GL (2018). Contextual and individual factors associated with dental services utilisation by Brazilian adults: a multilevel analysis. PLoS One.

[3] Kim HN, Han SJ, Jun EJ, Kim JB (2020). Factors Related to Oral Healthcare Service Utilization among Korean Adults Aged 25–79 Years. Int J Environ Res Public Health.

[4] Limpuangthip N, Purnaveja S, Somkotra T (2019). Predisposing and enabling factors associated with public denture service utilization among older Thai people: a cross-sectional population-based study. BMC Oral Health.

[5] Baker SR (2009). Applying Andersen's behavioural model to oral health: what are the contextual factors shaping perceived oral health outcomes?. Community Dent Oral Epidemiol.

[6] Silva AER, Langlois CO, Feldens CA (2013). Use of dental services and associated factors among elderly in southern Brazil. Revista Brasileira de Epidemiologia.

[7] Andersen RM (2008). National health surveys and the behavioral model of health services use. Med Care.

[8] Ship JA, Pillemer SR, Baum BJ (2002). Xerostomia and the geriatric patient. J Am Geriatr Soc.

[9] Lundegren N, Axtelius B, Isberg PE, Akerman S (2013). Analysis of the perceived oral treatment need using Andersen's behavioural model. Community Dent Health.

[10] Hajek A, König HH. Beyond symptoms: why do patients see the doctor? BJGP Open 2020;4(2). 10.3399/bjgpopen20X101088 PMC7330227 32430301

[11] Hajek A, Bock J-O, König H-H (2017). Association of general psychological factors with frequent attendance in primary care: a population-based cross-sectional observational study. BMC Fam Pract.

[12] Hajek A, Kretzler B, König H-H (2021). Factors associated with dental service use based on the Andersen model: a systematic review. Int J Environ Res Public Health.

[13] Lithuania population, 2021. https://www.worldometers.info/world-population/lithuania-population/. Accessed 3 May 2021.

[14] Petersen PE, Aleksejuniene J, Christensen LB, Eriksen HM, Kalo I (2000). Oral health behavior and attitudes of adults in Lithuania. Acta Odontol Scand.

[15] Janulyte V, Aleksejuniene J, Puriene A, Peciuliene V, Benzian H (2014). Current employment characteristics and career intentions of Lithuanian dentists. Hum Resour Health.

[16] Leinsalu M, Reile R, Vals K, Petkeviciene J, Tekkel M, Stickley A (2018). Macroeconomic changes and trends in dental care utilization in Estonia and Lithuania in 2004-2012: a repeated cross-sectional study. BMC Oral Health.

[17] Stangvaltaite-Mouhat L, Pūrienė A, Stankeviciene I, Aleksejūnienė J (2020). Erosive tooth Wear among adults in Lithuania: a cross-sectional National Oral Health Study. Caries Res.

[18] Stankeviciene I, Puriene A, Mieliauskaite D, Stangvaltaite-Mouhat L, Aleksejuniene J (2021). Detection of xerostomia, Sicca, and Sjogren’s syndromes in a national sample of adults. BMC Oral Health.

[19] World Health Organization (2013). Oral health surveys: basic methods - 5th edition.

[20] Laboratory for the study of stress, immunity, and disease. https://www.cmu.edu/dietrich/psychology/stress-immunity-disease-lab/scales/index.html. Accessed 30 May 2021.

[21] Lee EH (2012). Review of the psychometric evidence of the perceived stress scale. Asian Nurs Res (Korean Soc Nurs Sci).

[22] Wiriyakijja P, Porter S, Fedele S, Hodgson T, McMillan R, Shephard M, et al. Validation of the HADS and PSS-10 and a cross-sectional study of psychological status in patients with recurrent aphthous stomatitis. J Oral Pathol Med. 2020. 10.1111/jop.12991 31919894

[23] Alharbi H, Alshehry A (2019). Perceived stress and coping strategies among ICU nurses in government tertiary hospitals in Saudi Arabia: a cross-sectional study. Ann Saudi Med.

[24] Barros AJ, Hirakata VN (2003). Alternatives for logistic regression in cross-sectional studies: an empirical comparison of models that directly estimate the prevalence ratio. BMC Med Res Methodol.

[25] Official Statistics Portal. Urban and rural resident population by sex and age (5 years group) at the beginning of the year, 2018. https://osp.stat.gov.lt/web/guest/statistiniu-rodikliu-analize?hash=a947bb28-bf53-4b2b-986f-22bd9cb9cb12#/2018. Accessed 3 Aug 2021.

[26] Ghanbarzadegan A, Bastani P, Luzzi L, Brennan D (2021). Inequalities in utilization and provision of dental services: a scoping review. Systematic Reviews.

[27] National Health Insurance Fund under the Ministry of Health, 2021. https://ligoniukasa.lrv.lt/lt/veiklos-sritys/gyventojams-1/gydymo-ir-sveikatos-prieziuros-paslaugos/odontologines-paslaugos/pirmine-odontologine-pagalba. Accessed 23 Nov 2021.

[28] Government approves updated formula for pension increases, 2021. https://www.zmogausteises.lt/lietuva/vyriausybe-pritare-atnaujintai-pensiju-didinimo-formulei/. Accessed 6 Aug 2021.

[29] Can people afford to pay for health care? New evidence on financial protection in Lithuania. World Health Organization, 2018. https://www.euro.who.int/__data/assets/pdf_file/0005/372425/ltu-fp-report-eng.pdf. Accessed 7 Aug 2021.

[30] Manski R, Moeller J, Chen H, Widström E, Listl S (2016). Disparity in dental attendance among older adult populations: a comparative analysis across selected European countries and the USA. Int Dent J.

[31] Christensen LB, Rosing K, Lempert SM, Hede B (2016). Patterns of dental services and factors that influence dental services among 64-65-year-old regular users of dental care in Denmark. Gerodontology.

[32] EPd F, SGOd F, MdC M (2017). Factors associated with the use of dental care by elderly residents of the state of São Paulo, Brazil. Revista Brasileira de Geriatria e Gerontologia.

[33] Sakalauskiene Z, Maciulskiene V, Vehkalahti MM, Kubilius R, Murtomaa H (2009). Characteristics of dental attendance among Lithuanian middle-aged university employees. Medicina (Kaunas).

[34] Suominen-Taipale AL, Nordblad A, Alanen P, Alha P, Koskinen S (2001). Self-reported dental health, treatment need and attendance among older adults in two areas of Finland. Community Dent Health.

[35] Kiyak HA, Reichmuth M (2005). Barriers to and enablers of older adults' use of dental services. J Dent Educ.

[36] Official Statistics Portal. Number of practicing physicians, dentists and nurses per 10 000 population, 2017. https://osp.stat.gov.lt/web/guest/statistiniu-rodikliu-analize?hash=a947bb28-bf53-4b2b-986f-22bd9cb9cb12#/. Accessed 3 Aug 2021.

[37] Berlin V. Planning projections of dentist and dental specialist supply and demand in Lithuania until 2024 (doctoral dissertation).Vilnius: Vilnius University; 2016. Lithuanian.

